# Production and characterization of anthocyanin-rich beer from black wheat by an efficient isolate *Saccharomyces cerevisiae* CMS12

**DOI:** 10.1038/s41598-023-32687-1

**Published:** 2023-04-11

**Authors:** Arshpreet Singh, Saumya Singh, Sushil K. Kansal, Monika Garg, Meena Krishania

**Affiliations:** 1 Center of Innovative and Applied Bioprocessing (CIAB), Sector-81, Mohali, 140306 India; 2 Dr S S Bhatnagar University Institute of Chemical Engineering and Technology, Panjab University, Chandigarh, India; 3 National Agri-Food Biotechnology Institute (NABI), Sector-81, Mohali, 140306 India

**Keywords:** Biochemistry, Microbiology, Health care, Engineering

## Abstract

Beer is the world’s third most popular fermented beverage. It is typically made from malted barley. Tropical countries must import barley from temperate countries for brewing, which is an expensive process. Therefore, it is critical to investigate alternative possible substrates for beer production in order to meet the growing demand for high-nutritional-quality beer. The current study involves the creation of a fermented beverage from anthocyanin-rich black wheat with the help of yeast, *Saccharomyces cerevisiae* CMS12, isolated from fruit waste. Characterization (UV, HPLC, NMR, FTIR, and ICPMS) was then performed, as well as a comparative study with white (amber) wheat beer. Further, process parameters optimization included initial sugar concentration, inoculum size, and pH. Black wheat wort contained 568 mg GAE/L total phenolic content, 4.67 mg/L anthocyanin concentration, 6.8% (v/v) alcohol content, and a pH of 4.04. The sensory analysis revealed that black wheat beer was more acceptable than white wheat beer. The developed fermented beverage has enormous commercialization potential.

## Introduction

Around the world, there are numerous options for fermented foods and beverages, and the “fermentation” process is the underlying scientific method for potentially improving the nutritive value and digestibility of any substrate^1^. Microorganism-produced flavour improves the sensory acceptability of fermented foods^2^. Beer is the third most popular beverage after water and tea, with a global consumption of approximately 200 billion litres per year. Based on the ingredients and methods used in the brewing process, there are over 70 different types of beer^3^. The alcohol content of beer ranges from 0.5 percent to 15%^4^. Beer lowers the risk of cardiac arrest by lowering the concentration of low-density lipids and homocysteine, and it promotes kidney health^5^. Beer contains all the nutrients of cereal grains and hops rich in vitamins B, protein, minerals, dietary fibers, phenolics (antioxidants), ethanol, and prebiotic compounds^6^. The appearance of beer produced is affected by the strain of yeast used during the fermentation. The yeast affects the foam stability, head retention, haze formation and beer color^7^. Moreover, as yeasts utilize nutrients from the wort for growth and release the byproducts in it, changes in wort composition directly affect the flavor of beer^8^. The haziness in beer is due to the presence of proteins, polysaccharides, and polyphenols^7^. The mannoproteins derived from the cell walls of the yeasts help in minimizing haze formation and stabilization of foam. Protease enzyme is secreted by yeasts under unfavourable conditions, which degrades the proteins involved in formation and stabilization of beer foam. In addition, β-glucanases that hydrolyze β-glucan may be released by autolyzed yeasts, which result in reduction of viscosity and liquid drainage from the foam^9^. However, proper evaluation must be done before experimenting new yeast for brewing because every microbe is unique and can develop variations because of their interaction with different substances and conditions leading to various health concerns^10^. *Saccharomyces cerevisiae* is the common yeast in industries for brewing^11^. Numerous chemicals, such as organic acids, ketones, and esters, are produced when ethanol and carbon dioxide are combined, that have a significant influence on the sensory character of beer^12^. Brewers today are looking for alternative, more beneficial ingredients for beer production. Rice, saffron, wheat, barley, rye, maize, sorghum, potato hydrolysate, and low-to-moderate molasses were investigated as substrates for beer production^13,14^. Foods with high antioxidant content have been linked to health benefits, and anthocyanin-rich wheat (black wheat) is also known for its anti-obesity, blood sugar-lowering, and pre-biotic properties. Thus, beer production from such type of raw material can offer additional advantages to the consumers^15,16^. Popularity of beer produced from wheat malt has varied over the past years but lately, the demand for wheat beer has risen due to introduction of new brewing practices (craft and home brewing) and unavailability of barley in various regions^17^. However, wheat does not possess active enzymes responsible for production of sugars. So, to activate these enzymes and to ensure adequate levels of sugars are present for fermentation, malting of wheat is conducted. Malting is the main step of the brewing process. The purpose of malting is to promote the production of 11 hydrolytic enzymes in the grains^3^. Among the numerous varieties of wheat, black wheat is a hybrid crop prepared by crossing purple wheat and blue wheat which can serve as a potential substrate for beer production. This wheat is highly rich in anthocyanin content, protein, dietary fiber, and other nutrients etc.^18^. The current study used the biotechnologically developed black wheat, as a substrate to produce anthocyanin-rich beer from freshly isolated strain, *Saccharomyces cerevisiae* CMS12. This work did not use hops in beer production, so the antioxidant content and colour comes from the black wheat itself. The beer brewed with black wheat malt was compared to the beer brewed with white wheat malt. Further, the beer was characterized using UV, HPLC, NMR, FTIR, ICPMS, and a colour value. To the best of our knowledge of existing literature, no prior work has been reported on anthocyanin rich beer production from black wheat. The physicochemical properties and sensory profile of black wheat as a substrate for beer production are assessed in this study.

## Materials and methods

### Materials

#### Raw material

Two types of wheat namely, white (cv C306) and black (NABIMG-11; Ref.^19^) cultivated in the field of National Agri-Food Biotechnology Institute, Mohali, India in April 2022, were used for the beer preparation. Initially, both the wheat varieties with 60% relative humidity, were surface-sterilized with sodium hypochlorite and then kept at room temperature till further use.

#### Chemicals

The microbial media constituents were procured from HiMedia Laboratories, Mumbai, India. As a control yeast, *Saccharomyces cerevisiae* was purchased from Fermentis Company in Lesaffre, France. Analytical grade reagents, indicators, solvents, enzymes, and chemicals were ordered from Merck, Sigma Aldrich, USA. For all experiments, borosilicate glassware and double-distilled water were used.

### Methods

#### Screening, isolation, and identification of yeast for beer production

Fruit by-product samples (apple, grapes, and banana) were collected in sterile containers and refrigerated at 4 °C until used to isolate ethanol-producing yeasts. Isolation was accomplished through serial dilutions up to 10^−6^ using the spread plate method and glucose yeast extract (GYE) agar plates for 48 h at 30 °C. The desired isolates based on their ethanol producing potential were analyzed and selected using HPLC. The colonies were introduced into a flask of 250 mL containing 50 mL GYE broth and kept at 28 °C with 150 rpm for ethanol production^20^. The samples were collected over a 6 h period and analyzed using High Performance Liquid Chromatography (HPLC) (Agilent, HiPlex, California, USA), with a RID detector set to 55 °C and an Agilent HiPlex H column (300 mm × 7.7 mm, 8 m) set to 60 °C. The mobile phase (5 mM H_2_SO_4_) flow rate was 0.7 ml/min. Before HPLC, the mobile phase was degassed and filtered through a 0.22 µm nylon membrane filter (Millipore, MA). The yeast with the highest ethanol yield under mesophilic conditions was chosen. An inverted light microscope (40X) was used to examine the morphology of promising strains (Nikon Eclipse TS2, USA) and the culture was identified at a facility of Institute of Microbial Technology, Chandigarh.

#### Optimization of fermentation parameters for ethanol production

Fermentation parameters such as substrate (glucose) concentration (40–80 g/L), inoculum size (2–12%), and pH (4.5–7) were screened using the one-factor-at-a-time (OFAT) technique to maximize ethanol production by isolated yeast strains from fruit wastes. All trials were carried out at 28 °C in microaerophilic conditions with shaking at 150 rpm^21^. The broth was recovered after centrifugation (6000 *rpm*, 10 min), and residual sugars and ethanol were measured using HPLC.

#### Extraction of fermentable sugars from wheat

Malt was made by steeping, germination, and kilning wheat grains^22^. To summarize, 250 g of wheat was soaked in 1000 mL of water for 6 h at 16 °C to increase the moisture content from 12 to 40%. For two days, the grains were covered in germination paper and left at room temperature. Germinated grains were kilned to make grist, which was then mashed. The grist was mashed with 3:1 deionized water (water:grist) and mixed at a speed of 150 rpm before being heated at 45 °C for 1 h. With constant mixing at 150 rpm, the temperature was raised to 52 °C for 15 min, 65 °C for 45 min, and 75 °C for 15 min. After mashing, the filtrate was centrifuged for 20 min at 10,000 *rpm* and boiled for 30 min at 100 °C^23^. The cooled precipitates were filtered through filter paper, and the filtrate (wort) was fermented.

#### Inoculum preparation for fermentation

At 28 °C, the yeasts were cultured for 24 h on GYE-agar plates. A colony loop was introduced into 50 mL of media (GYE broth) in a 250 mL flask prior to fermentation. Incubate for 24 h at 28 °C in a shaker (Innova42, New Brunswick Scientific, CT, USA) at 150 rpm^22^.

#### Fermentation of prepared wort for beer production

Fermentation experiments were conducted with 50 ml of wort in 250 ml flasks. The fermentation media was inoculated with a 24 h old yeast inoculum and kept at 28 °C with 150 *rpm* for 120 h^23^. After the fermentation process was completed, the broth cells were separated by centrifuging for 15 min at 6000 *rpm*^21^. The consumption of sugar (glucose, maltose) and ethanol formation was monitored by taking, periodic samples every 6 h and analyzed using HPLC.

#### Kinetics of beer production

To investigate the kinetics of prepared black wheat beer using a non-linear regression model and the “LABFIT” tool (V 7.2.50, Campina Grande, Brazil). The 95% confidence interval was used to compare logistic model predictions to experimental production rates (CI). The kinetic factors a, b, and c to produce black wheat beer were also calculated.

#### Wheat fermentation and its sensory evaluation

The beer was prepared in 2L batches and its physicochemical and organoleptic properties were investigated. Under the same conditions, 1 kg of wheat (black and white) was used to make malt. HPLC was used to determine sugar and ethanol levels during fermentation. The beer was chilled after being pasteurized at 63 °C for 30 min. Semi-trained professionals (n = 9) from Mohali, India’s Center of Innovative and Applied Bioprocessing evaluated the sensory parameters of beer on a nine-point hedonic scale for appearance, taste, colour, aroma, and overall acceptability^24,25^. Whereas 9-like extremely, 8-like greatly, 7-like moderately, 6-like slightly, 5-like neither, 4-dislike slightly, 3-dislike moderately, 2-dislike highly, and 1-like extremely.

#### Characterization of beer

##### Evaluation of physico-chemical properties

The pH (pH meter, Mettler Toledo, Mumbai, India), total soluble solids (TSS), and titrable acidity of the prepared beer samples were determined^26^. HPLC was used to determine the amount of ethanol and sugars. Inductively Coupled Plasma Mass Spectrometry (ICP-MS) was used to examine the minerals (7800 ICP MS, Agilent Technologies, USA). Total phenol content was determined using Kumari et al.^27^ methodology at 650 nm with a spectrophotometer (UV3000 +, Lab India, Delhi, India). The total monomeric anthocyanins (TMA) content was determined using two different wavelengths of 520 and 700 nm^28^. Color was determined using EBC (European Brewing Convention) units at 430 nm^29^.

##### NMR (Nuclear Magnetic Resonance)

Samples were dissolved in deuterated dichloromethane in 5 mm fully dry NMR tubes. For NMR spectroscopy, samples were degassed in ultrasonicator for 10 min before analysis. Tetramethylsilane was utilized as an internal standard^30^. For the characterization of organic molecules, measurements with a relaxation delay of 6 s were carried out in a Bruker Advance 300 spectrometer operating at a magnetic field strength of 400 MHz^28^.

##### FTIR (Fourier- Transform Infrared Spectroscopy)

Samples were analyzed using an FTIR coupled with ATR analysis (Agilent model: Cary 660 Series) with 4000–600 cm^−1^ scale and a variation of 4 cm^−1^ in order to quantify the change in chemical structure. A blank ATR cell was utilized to measure the background of the samples. In order to compare black and white wheat beers, the absorbance strength of each spectrum was observed.

#### Analytical procedure

For the quantitative measurement of monosaccharides and ethanol obtained during the production process, high performance liquid chromatography (HPLC) (Agilent, HiPlex, Santa Clara, California, USA) was used. For determination, a refractive index detector (RID) was used at 55 °C. The mobile phase was 5 mM H_2_SO_4_ at a flow rate of 0.7 ml/min in an Agilent HiPlex H analytical column (300 mm 7.7 mm, 8 m) operating at 60 °C.

#### Statistical analysis

A triplicate of each sample was conducted. For the purpose of representing pertinent results, the mean and standard deviation of the data values are used. Data were subjected to analysis of variance by ANOVA with statistical significance (P < 0.05) and compared by using the least significant difference (LSD) test. A significance level of p value < 0.05 is used to reflect the results. All statistical analyses were performed using software (IBM-SPSS, Version-28, Armonk, New York (NY), USA).

### Ethics declaration

The plant collection and use were in accordance with all the relevant guidelines of National Agri-Food Biotechnology Institute (NABI), Mohali.

## Results and discussion

### Identification and taxonomic study of ethanol producing yeasts

**Figure 1:**
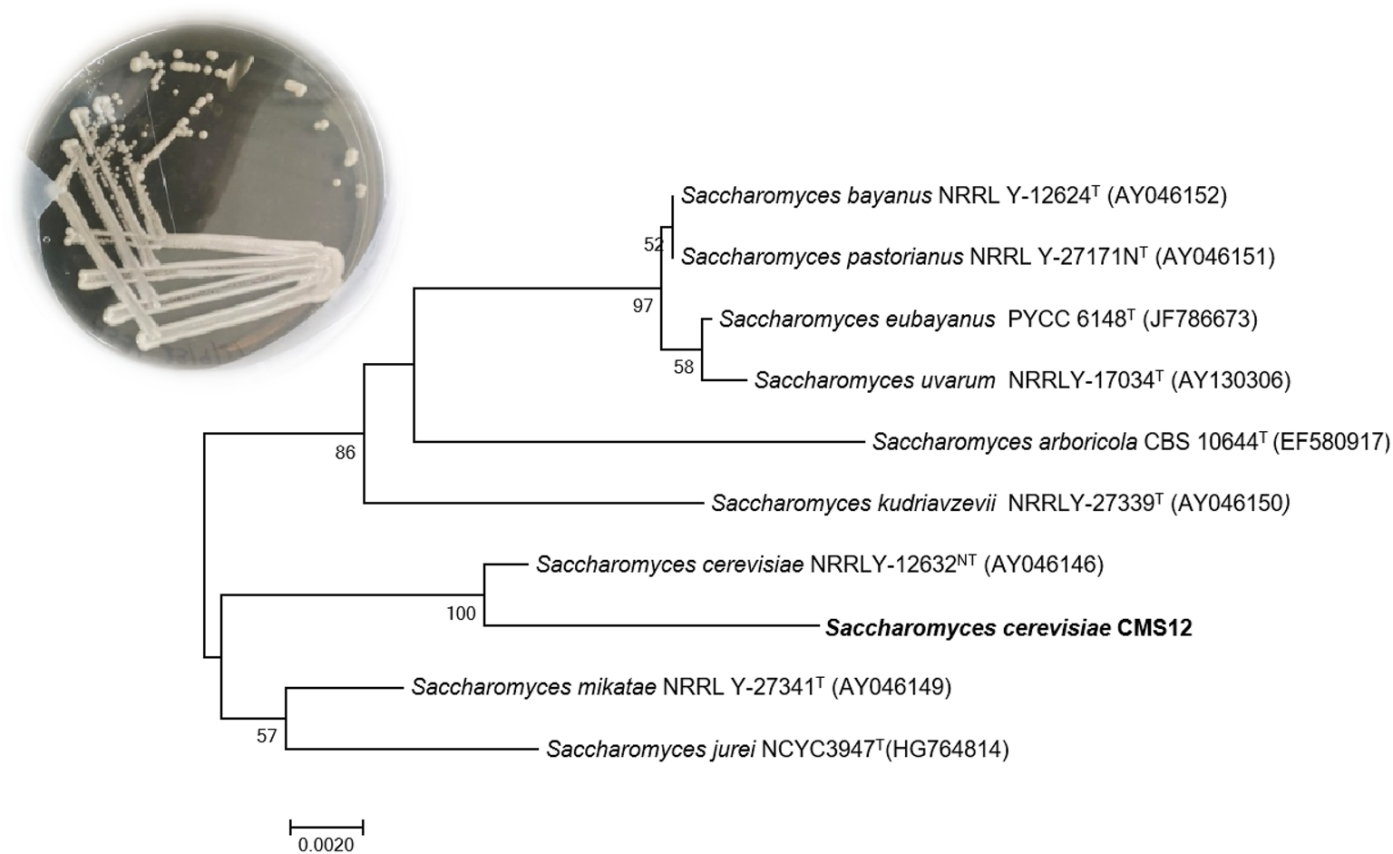
Phylogenetic tree based on ITS and D1/D2 gene sequence comparison showing the position of *S. cerevisiae* CMS12 and other related species of genus *Saccharomyces*.

Out of 25 isolates that were screened, two potential yeasts, designated as C1 and C2 were selected based on their ability to give desirable ethanol yield. On an agar plate, both the isolates (C1 and C2) produced small creamy-white colonies (~ 5 mm) that were raised and circular. The cells observed under a 40X microscope were ovoid in shape and showed signs of budding as shown in Supplementary Fig. B. Sequence of the D1/D2 domain of 26S rRNA and 5.8S-ITS rDNA analyzed with GenBank database revealed an evolutionary relationship with other closely related *Saccharomyces cerevisiae* strains and 100% homology with *Saccharomyces cerevisiae* NRRLY-12632^NT^ (AY046146), with which the test shares common ancestor. Therefore, post taxonomic identification, the strain C2 was named by its scientific nomination together with lab code as *Saccharomyces cerevisiae* CMS12 (Fig. 1). Similarly, the strain C1 was identified as *Saccharomyces cerevisiae* CMS 11. The morphological characteristics of isolated yeasts were the same as those observed for isolated *Saccharomyces cerevisiae* from sugar cane molasses^31^. In this study, both the identified strains were referred to as C1 and C2 for ease of reference.

### Optimized ethanol production on synthetic media

**Figure 2:**
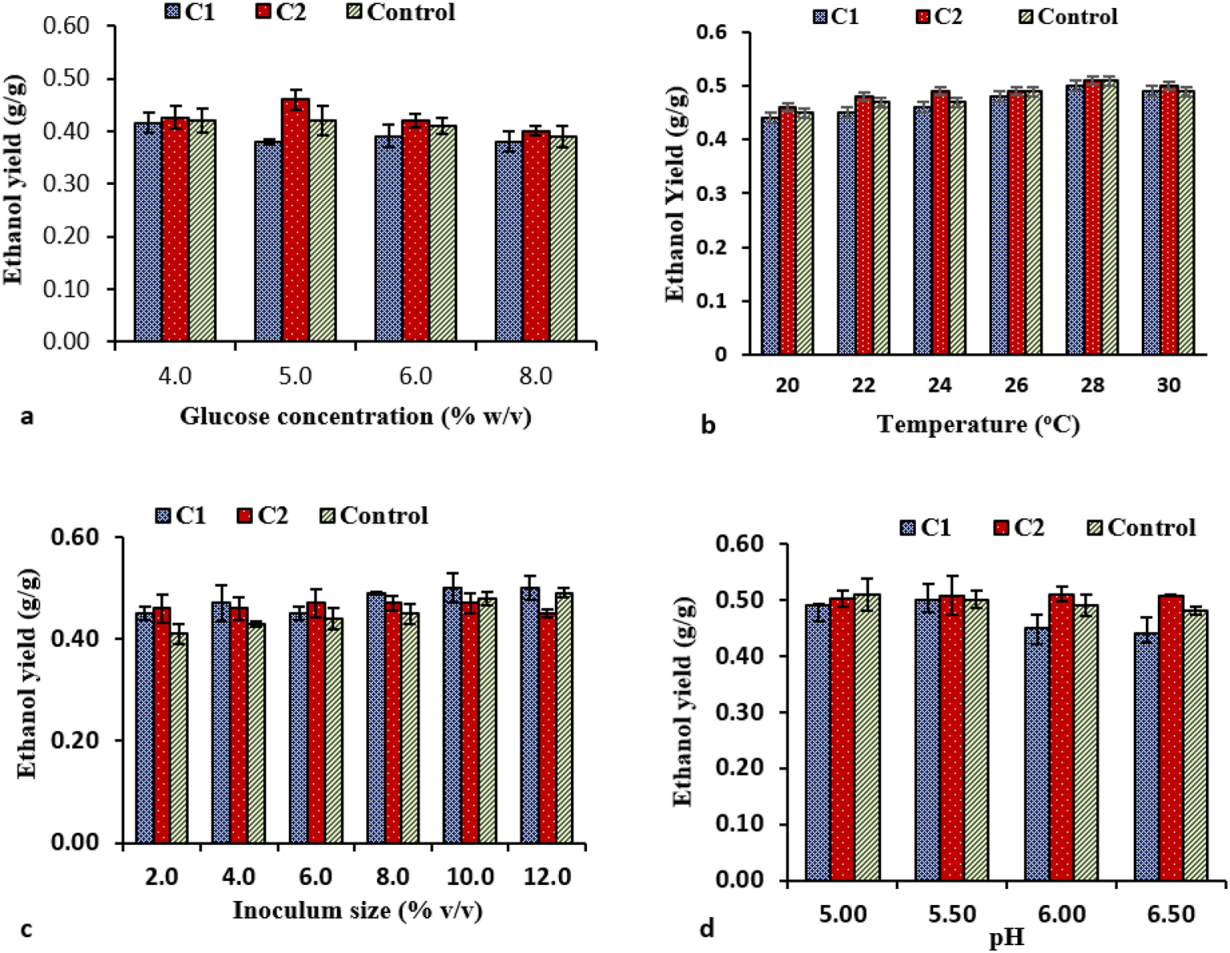
Fermentation process parameters optimization using control strain and strains isolated in this study (C1, C2) (**a**) Glucose concentration (**b**) Temperature (**c**) Inoculum size (**d**) pH. C1: *Saccharomyces cerevisiae* CMS11; C2: *Saccharomyces cerevisiae* CMS12; Control: commercial culture of *Saccharomyces cerevisiae.*

The screened and isolated yeast strains (C1 and C2) were optimized to produce ethanol on synthetic media as schematically represented in Supplementary Fig. A. Figure 2a shows that ethanol production decreases as sugar concentration rises this is due to increase in osmotic pressure and represses oxidative pathways. Whereas, at low substrate concentrations, yeast starved and production dropped^14,32^.

Inoculum size of 10% v/v for C1 and 8% for C2, yielded 50% and 47% ethanol conversion efficiency from substrate, respectively. No significant difference in ethanol conversion efficiency was observed when compared to control yeast with 12% or 10%v/v inoculum size (Fig. 2b). Therefore, 10% inoculum size was decided for further study. Wilkins et al.^33^ produced optimized ethanol using *Saccharomyces cerevisiae* with 10% v/v inoculum in 72 h fermentation. Increasing the inoculum size did not improve fermentation because it led to substrate exhaustion as reported previously^34^.

The isolated yeast strains resulted in optimum ethanol production at pH 5.5. The production decreased as the pH increased (6.5) (Fig. 2c). Therefore, pH 5.5 was decided as optimal for C1 and C2 strains for ethanol production as reported previously^35^.

Temperature affects yeast growth and volatile organic levels. At 28 °C, yeast growth and alcohol production are faster than at lower temperatures. Both C1 and C2 showed similar observations (Fig. 2d). Therefore, 28 °C decided as optimal. Previous observations were similar^36^.

### Fermentation of black wheat wort and kinetics of potential strain

**Figure 3:**
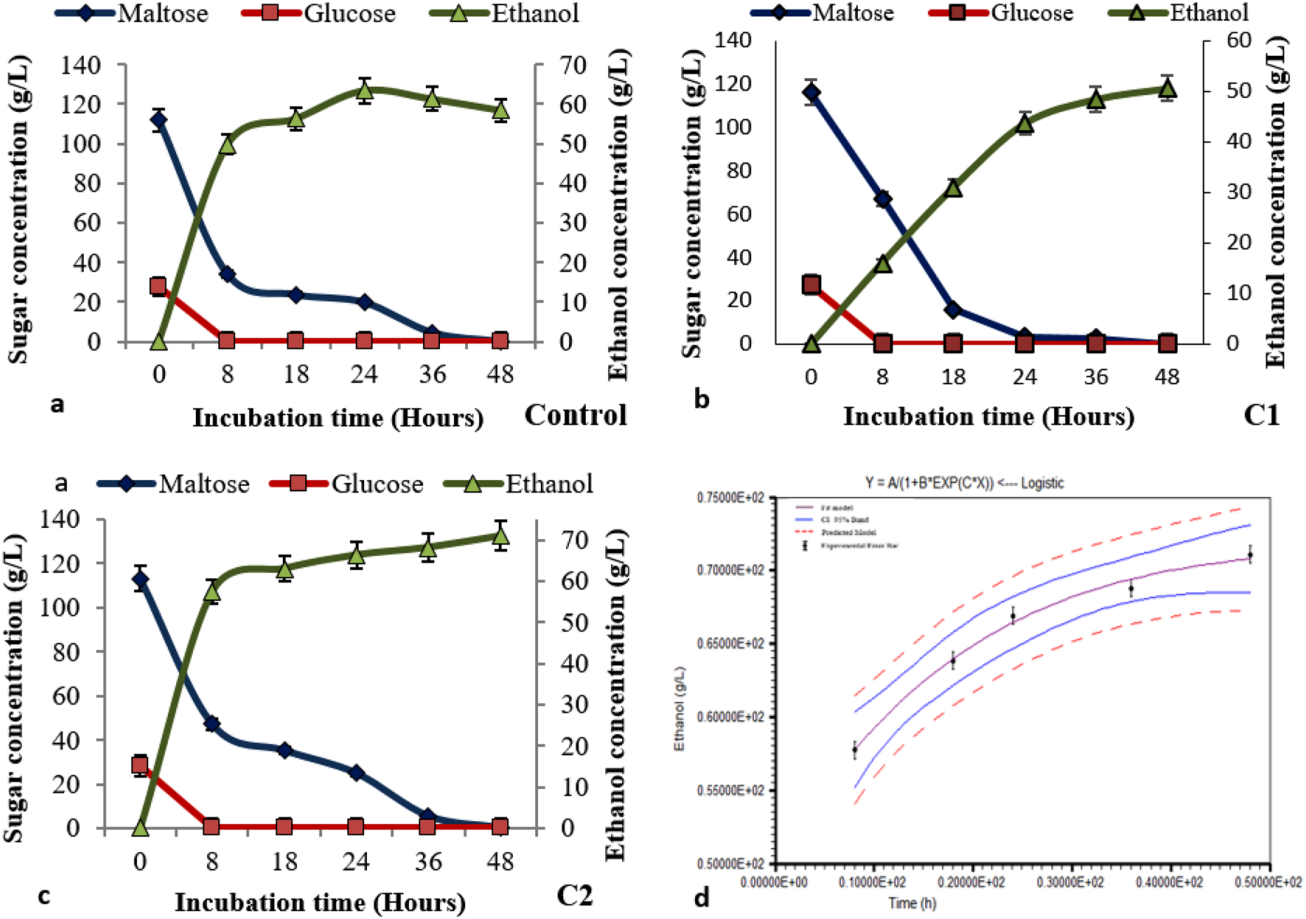
Time lapse changes in the maltose, glucose, and ethanol concentrations of black wheat wort after fermentation with different yeast strains (**a**) Standard yeast (*Saccharomyces cerevisiae*) as control (**b**) Strain C1 and (**c**) Strain C2 (**d**) Kinetic modelling of potential strain C2.

In the black wheat wort fermentation, the glucose consumption was substantially faster than maltose consumption. During the initial 8 h of fermentation, C2 yeast strain completely digested glucose and showed maximum production of ethanol (71.98 g/L), that was higher than the standard yeast (Fig. 3a–c). After 48 h of fermentation, the maltose concentration gradually declined until it was completely depleted.

With the application the logistic model^37^ for beer production and C2 as a potential strain. The curve closely fit to projected band with 95% confidence interval. R^2^ = 0.99 (Fig. 3d).

Extrapolated the kinetic parameters from the below equation:

$${\text{P}}\, = \,{\text{a}}\,/\,\left\{ {{1}\, + \,{\text{b}}\,{\text{exp}}\,\left( {{\text{c}} * {\text{x}}} \right)} \right\},$$

P = a/{1 + b exp (c*x)}.where P = Ethanol production (g/L), a = Maximum ethanol concentration = 71.95 ± 02 g/L, b = Fermentation time = 42.2 ± 0.2 h, c = Conversion rate = 1.67 ± 0.1 g/Lh.

Statistically significant values were identified by the one-way ANOVA test with a p value < 0.05.

### Physico-chemical evaluation of beer

**Figure 4:**
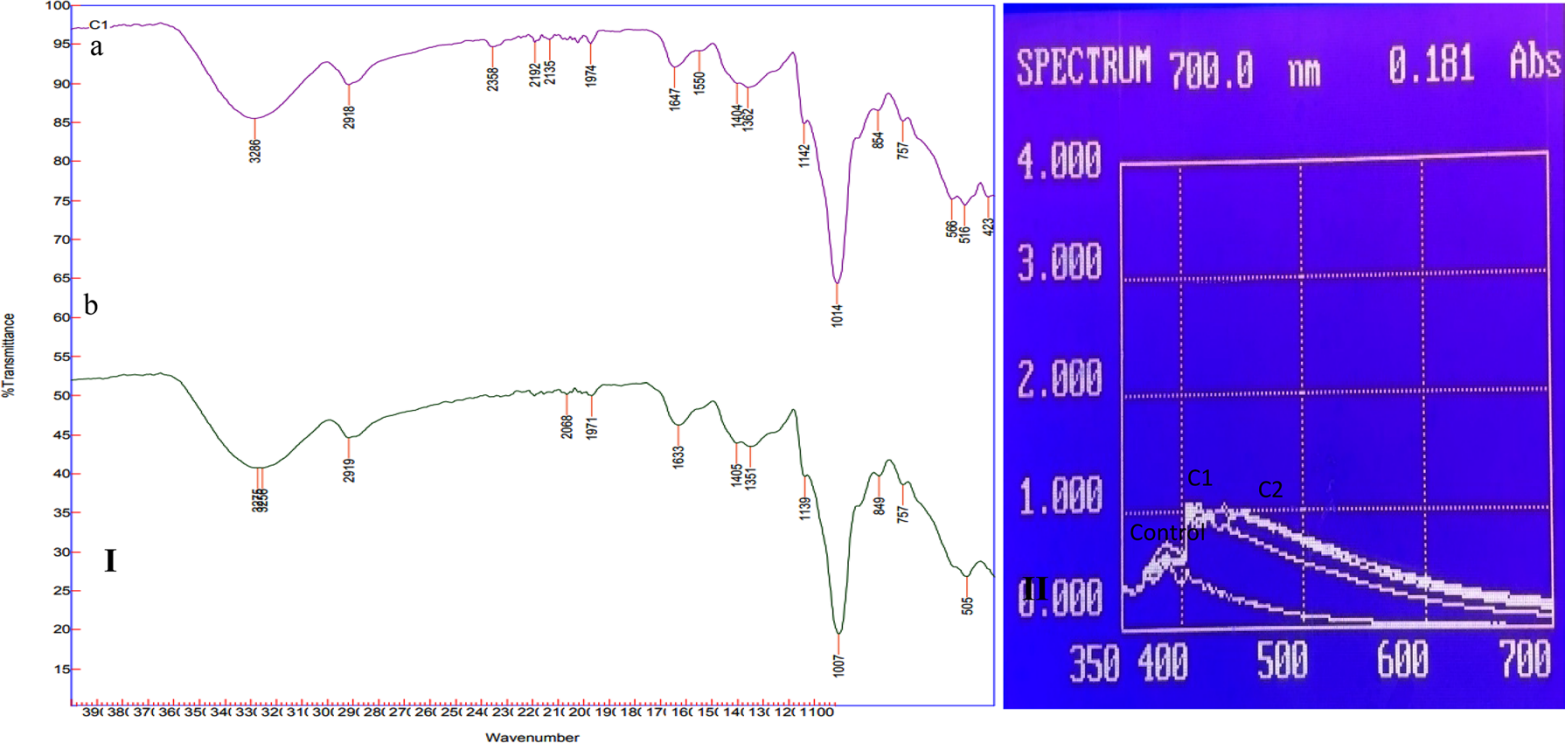
**(**I) FTIR of (**a**) White wheat beer and, (**b**) Black wheat beer, (II) UV Spectra showing anthocyanin in the black wheat beer production using C1, C2 and control yeast strains.

The black wheat beer prepared from commercial strain of yeast *S. cerevisiae* taken as control showed higher pH (4.7) than both isolated strains. The C1 strain produced beer with least acidity (0.12) and pH (4.0). The C2 strain produced beer with acidity (0.28) and low pH (4.0). However, the black wheat beer, from C2 strain produced higher alcohol than others. The C1 strain contained 6.52% alcohol (v/v), while the C2 and control strains contained 7% and 6.41% alcohol, respectively (Table 1). The published literature indicates that beer from different malts’ have 3.50–12% alcohol, 4.0–5.0 pH, and 0.1–0.3 acidity^38,39^. The observed data agrees with the published literature. However, the beer produced from three strains showed similar color. The color analysis of brewed black wheat beer with C1, C2, and control yeast revealed 22.95, 21.45, and 22.72 EBC, respectively. The color is due to anthocyanin (Fig. 4I), as reported in fruits with color ~ 25.8 EBC^40^. Whereas, increased phenolic content was observed in isolated strains compared to control, C2 strain is highest. It was 609.37, 613.12, and 568.00 mg GAE/L in C1, C2, and control beer, respectively. The ale beer has 563 mg GAE/L^41^. High-phenolic beers have a longer shelf life, better taste, and fragrance than low-phenolic beers^42^.

**Table 1:** Physicochemical characteristics of beer produced from black wheat by isolated yeast and control yeast (*Saccharomyces cerevisiae*).

Yeast strain	Colour (EBC)	pH	Total acidity	TSS (ºB)	Reducing sugars (g/100 ml)	Anthocyanin content (mg/L)	Alcohol content (%)
Isolated strain C1	22.95^a^ ± 0.34	4.4^a^ ± 0.03	0.12^a^ ± 0.05	12.80^ab^ ± 0.41	0.23^a^ ± 0.06	5.67^a^ ± 0.05	6.52^a^ ± 0.48
Isolated strain C2	21.45^b^ ± 0.28	4.0^b^ ± 0.05	0.28^b^ ± 0.02	13.10^a^ ± 0.22	0.20^a^ ± 0.05	6.43^b^ ± 0.03	7.00^a^ ± 0.35
Standard strain (Saccharomyces cerevisiae)	22.72^a^ ± 0.17	4.7^c^ ± 0.02	0.24^b^ ± 0.03	12.20^b^ ± 0.32	0.22^a^ ± 0.04	4.67^c^ ± 0.04	6.41^a^ ± 0.44

*TSS* total soluble solids.

Values are mean of three replicates ± standard deviation (p < 0.05).

Values with different superscript roman letters (a and b) in the same column are significantly different according to the Tukey’s range test (p < 0.05).

Total wort monomeric anthocyanin content (TMA) before and after boiling was 10.52 mg/L and 7.85 mg/L respectively. This drop may be due to heat-sensitivity of anthocyanins^43^. TMA in black wheat beer with isolated strains was higher than control being highest in C2 strain. The TMA for C1, C2, and control yeast were 5.67, 6.43, and 4.67 mg/L respectively. Yeasts’ anthocyanin absorption during fermentation may affect results. Similar observations have been reported previously for black rice wort^44^. As the beer produced by the strain C2 showed highest alcohol production, phenolic and anthocyanin content it was selected for further experimentation. The final anthocyanin yield and alcohol content obtained in our work was higher than the study on beer production using sweet potato by *Saccharomyces cerevisiae, which produced beer with 3.5 pH,* 5.10 mg/100 mL anthocyanin and 3.77% alcohol content^39^. Another study by Piraine et al.^45^ using S. cerevisiae WLP001 beer produced with pH 4.30 and alcohol content 3.57%. Also, the low pH in case of strain C2 also indicated its applicability in production of sour beers. Another study reported an ethanol content of 5.37% (v/v) on *S. cerevisiae* fermented beer with Lubelski and 5.22% (v/v) in beer with Marynka hops^46^. However, some studies also succeeded in increasing the alcohol content up to 9.6–10.46% by providing adequate conditions during the fermentation process and by optimizing fermentation media^47^. This implies, that apart from the strain’s potential, the alcohol content also depends upon the types of hops used. However, from the aspect of using strain in the brewing industry, resistance to higher concentration to ethanol is not the main criteria as opposed to the percentage ethanol produced.

### Higher scale beer production and its sensory evaluation

Black wheat and white wheat beer were produced at 2 L scale. Better physicochemical properties were observed in black wheat beer compared to white wheat beer including alcohol content, EBC and acidity (Table 2). The beer’s physicochemical properties influence consumer acceptance. The data presented in Table 3, represents the mean sensory score including appearance/color, taste, flavor, and overall acceptability. The overall acceptability for black wheat beer was slightly high than white wheat beer. The color variation of beers was noticeable; the black wheat beer has an orange-red color whereas the white wheat beer has a pale gold color. In the mineral contents (Supplementary Table A), the potassium and the magnesium were higher 1661.17 ppm, 486.50 ppm in black wheat beer than control white wheat beer 526.13 ppm, 126.80 ppm respectively. In addition, the calcium and zinc were also higher in black wheat beer 20.97 ppm, 1.62 ppm than the white wheat beer 8.33 ppm, 0.22 respectively^48^.

**Table 2:** Physicochemical attributes of beer produced from control wheat and black wheat.

Parameters	Control wheat beer	Black wheat beer
Alcohol content (%)	3.67^a^ ± 0.58	6.80^b^ ± 0.45
pH	4.20^a^ ± 0.05	4.04^a^ ± 0.03
Reducing sugars (g/100 ml)	0.17 ± 0.03	0.23 ± 0.04
Brix	9.00^a^ ± 0.48	13.00^b^ ± 0.32
Acidity (%)	0.33^a^ ± 0.05	0.27^a^ ± 0.02
EBC	7.30^a^ ± 0.36	21.45^b^ ± 0.30

*EBC* color determined by European brewing convention units at 430 nm.

Values are mean of three replicates ± standard deviation (p < 0.05).

Values with different superscript roman letters (a and b) in the same column are significantly different according to the Tukey’s range test (p < 0.05).

**Table 3:** Sensory scores of beer produced from black wheat and control white wheat.

Sensory attributes	Beer (Black wheat)	Beer (White wheat)
Color/Appearance	7	6.5
Flavor	7	7
Mouthfeel	7.5	7
Overall Acceptability	7	6.5
Total score	28.5	27

### FTIR (Fourier- Transform Infrared Spectroscopy)

The portion of the spectrum below 1500 cm^−1^ is difficult to relate to a specific molecular vibration in a mixture as complex as beer, because each molecule creates a distinct absorption pattern in this region of the spectrum. However, the C–O stretching was observed because of dextrin in black wheat beer is at 1007 cm^−1^ and in white wheat beer at 1014 cm^−1^. A series of spectral bands placed below 1500 cm^−1^ correspond to the vibration of C–C and hydroxyl groups in carbohydrates and ethanol. In both white and black wheat beers, the ethanol absorbs at ~ 2919 cm^−1^; this wavelength is similar to the asymmetric stretching band of the methyl group. Whereas, the O–H stretching was observed in the range of band 3200 cm^−1^–3300 cm^−1^ in both beers, because water and ethanol molecules can form hydrogen bonds with one another. The band range from 1600 cm^−1^ to 1900 cm^−1^ are labeled with stretching C = O^49^ and is connected to the myriad of different chemical components that can be found in beer, such as vitamins and soluble solutes^50^.

### UV–Vis spectroscopy

UV–Vis spectroscopy has been widely used to identify anthocyanins. When carefully analyzed, the spectrum can tell you useful things about how anthocyanins are put together. Majorly the UV–Vis data are still useful to confirm the general structure of anthocyanins and to describe the unsaturated and functional groups in the different parts of the anthocyanin structure. In general, anthocyanins show a typical absorption pattern on the UV–Vis spectrum as shown in Fig. 4II. Most of the time, the absorption maximum (λ_max_) in the visible range is between 510 and 520 nm, followed by a curve between 400 and 450 nm. It is easy to see that C1, C2, and the control have anthocyanins in their UV–Vis spectra. A hump is also seen between 400 and 450 nm followed pattern in visible range 500–600 nm. The size of this hump depends on how many sugar molecules are attached to the anthocyanidin moiety. In general, the structure of anthocyanin has a fully delocalized -conjugated system that makes it stable. Previous studies have shown that anthocyanins follow a similar pattern^51^.

### NMR (Nuclear Magnetic Resonance)

**Figure 5:**
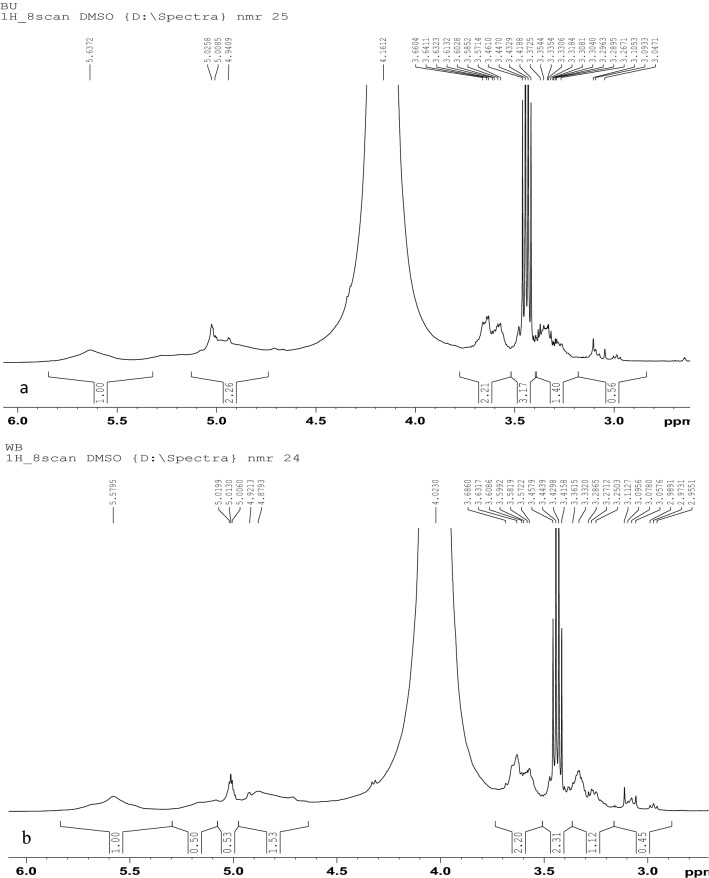
NMR of (**a**) White wheat beer and (**b**) Black wheat beer.

1H NMR spectra of black wheat and white wheat beer are shown in Fig. 5. Overall, similar peaks patterns were observed in both the black and white wheat beer. Whereas, the highly responsive region near around 3.5 ppm represents the organic acids like citric acid, succinic acid, pyruvic, and acetic acid in both the samples^52^. Moreover, dextrins and sugars were observed at 5 ppm in the spectrum^53^. Overall, similar peaks patterns were observed in both the black and white wheat beer along with the reported literature^30,52^.

## Conclusion

Brewers are looking for ways to make their products more lucrative as demand for beer continues to rise. The findings of this study suggest that it is possible to create a new beer using black wheat with high anthocyanins and minerals content. The isolated strain C2 (*Saccharomyces cerevisiae* CMS12) produced black wheat beer with higher levels of alcohol (7% v/v) and anthocyanin constituents (6.43 mg/L). Black wheat beer had a higher acceptability than white wheat beer, according to sensory evaluation. Although, the developed black wheat beer, besides being inherently rich in nutrients, is also a rich source of anthocyanins and has potential to impart several direct and indirect health benefits. However, it should be consumed in moderation and responsibly as excess of everything is bad. As a fermented beverage, the newly developed black wheat-based beer has significant marketability potential.

## Supplementary Information

Supplementary Information.

## Data Availability

The datasets used and/or analyzed during the current study available from the corresponding author on reasonable request.
