# Supplementary material for: Production and characterization of anthocyanin-rich beer from black wheat by an efficient isolate Saccharomyces cerevisiae CMS12
Source: Sci Rep. 2023 Apr 11;13:5863. doi: 10.1038/s41598-023-32687-1 (PMC10090066; doi:10.1038/s41598-023-32687-1)
Supplement: Supplementary file 1 — Supplementary Information. [file 41598_2023_32687_MOESM1_ESM.docx]

Supplementary file

**
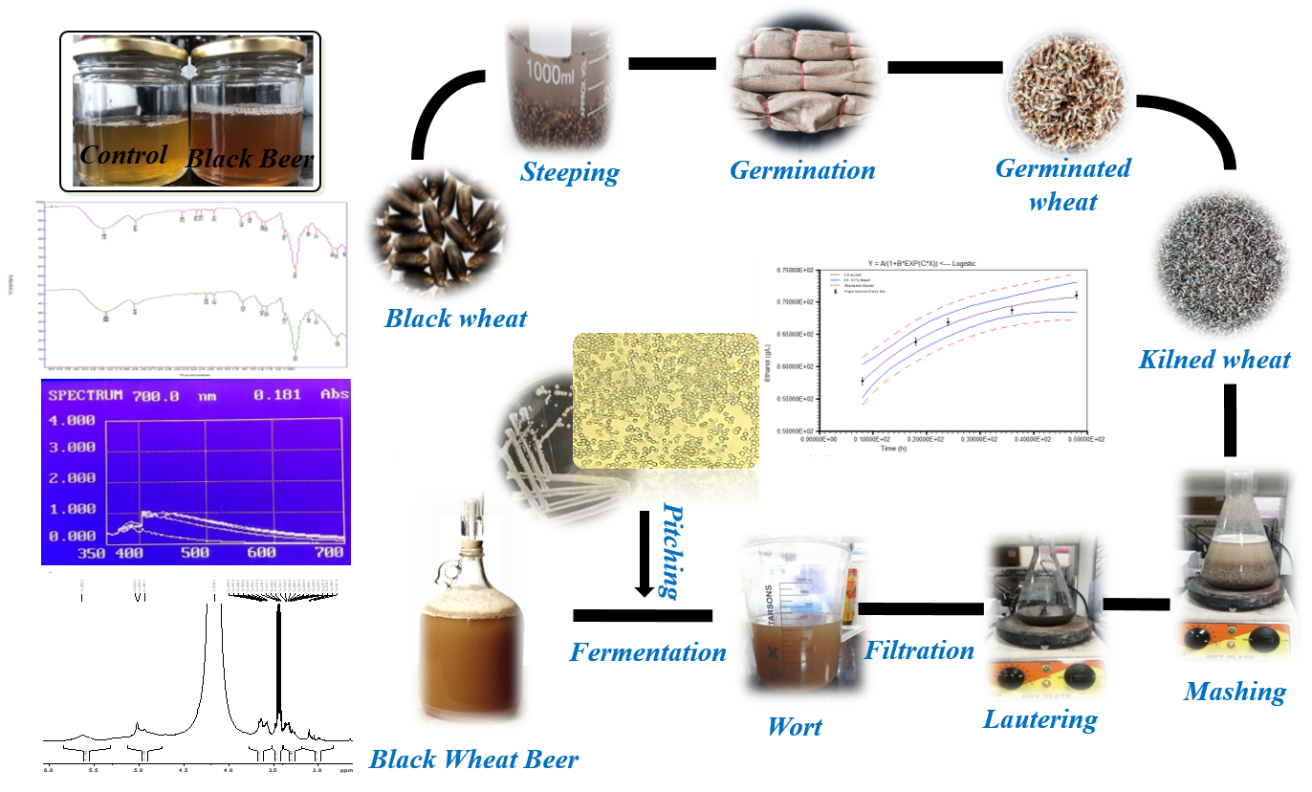
**

**Supplementary Fig. A** Schematic representation of anthocyanin rich beer production from black white using the isolated strain *Saccharomyces cerevisiae* CMS12


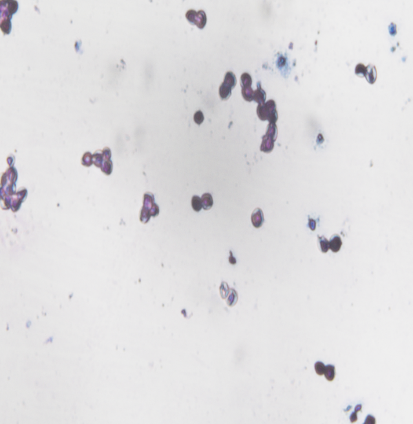

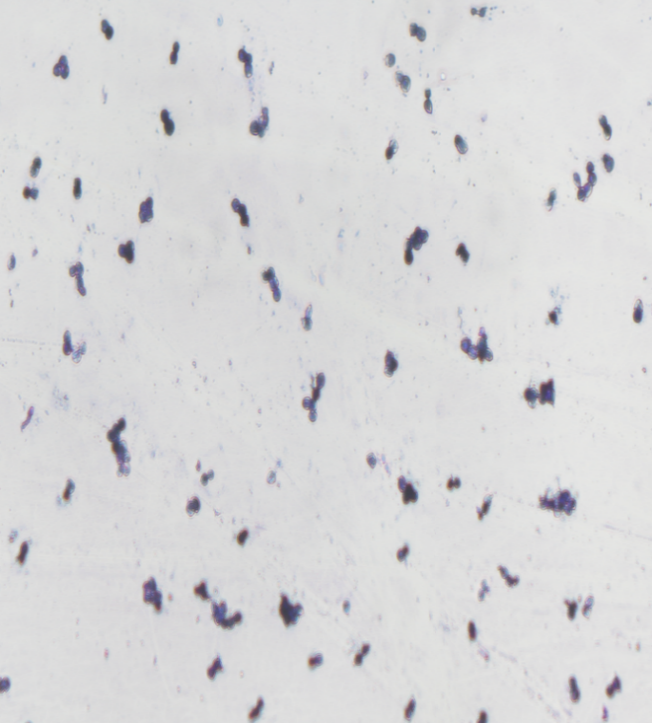

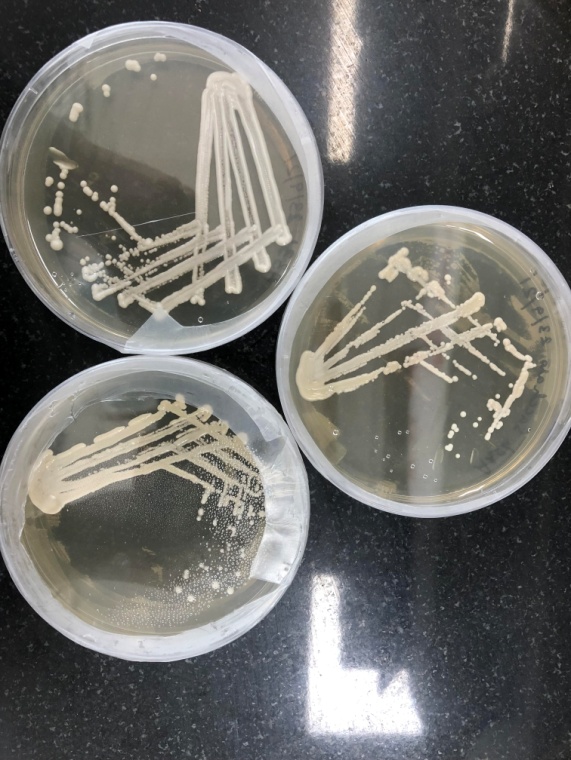


C2

C1

C1

C2

C2

C2

C1

**Supplementary Fig. B** Streaked colonies of newly isolated yeasts C1 and C2 and their microscopic evaluation

**Supplementary Table A.** Mineral composition of beer produced from black wheat and white wheat

| Minerals (ppm) | | Black wheat beer | | Control beer (White wheat) | |
| --- | --- | --- | --- | --- | --- |
| Sodium | | 187.88 | | 111.55 | |
| Magnesium | | 486.50 | | 126.80 | |
| Chlorine | | <LOD | | <LOD | |
| Potassium | | 1661.17 | | 526.13 | |
| Calcium | | 20.97 | | 08.33 | |
| Iron | | 00.036 | | 00.079 | |
| Zinc | | 01.62 | | 00.221 | |
